# COGNITIVE IMPAIRMENT, FALLS, AND DEMENTIA: NOVEL APPROACHES FOR EARLIER DETECTION AND MORE EFFECTIVE INTERVENTION

**DOI:** 10.1093/geroni/igad104.1259

**Published:** 2023-12-21

**Authors:** Kieran Reid, Helena Blumen, Joe Verghese

## Abstract

This symposium will discuss novel digital approaches for the remote assessment of cognitive status and detection of fall risk in older adults. We will then describe several emerging digital exercise, ballroom dancing, and other non-invasive intervention strategies targeted for vulnerable older populations at increased risk for falling, or developing cognitive impairment and dementia. Collectively, we posit that these innovative detection and intervention strategies hold significant potential as more effective and scalable approaches for identifying cognitive impairment, preventing falls, and mitigating cognitive decline in high-risk older adults. In presentation 1, Roque et al. examines the utility of ecological momentary cognitive assessment and its potential as a digital biomarker of cognitive impairment in community-dwelling older adults. In presentation 2, Mahoney et al. discusses the development and clinical potential of a novel digital health app, CatchU®, that remotely captures visual-somatosensory integration performance to detect fall propensity in older adults. In presentation 3, Bajdek et al. describes the feasibility of a 12-week digitally delivered home-based fall prevention exercise program for older adults with elevated fall risk. In presentation 4, Blumen et al. report preliminary efficacy and mechanistic findings from a randomized trial of social ballroom dancing in older adults at increased risk for dementia. In presentation 5, Reid et al. describes the feasibility of a real-world community-level physical activity intervention for older adults with motoric cognitive risk syndrome. The discussant, Dr. Verghese, will integrate the key findings from these studies, discuss their theoretical and methodological contributions, and consider future research directions.

